# Determinants of accepting non-invasive ventilation treatment in motor neurone disease: a quantitative analysis at point of need

**DOI:** 10.1080/21642850.2013.848169

**Published:** 2013-11-01

**Authors:** Rosanna Cousins, Hikari Ando, Everard Thornton, Biswajit Chakrabarti, Robert Angus, Carolyn Young

**Affiliations:** ^a^ Department of Psychology, Liverpool Hope University, Liverpool, UK; ^b^ Walton Centre for Neurology and Neurosurgery, Liverpool, UK; ^c^ Department of Psychology, University of Liverpool, Liverpool, UK; ^d^ Chest Centre, Aintree University Hospital, Liverpool, UK

**Keywords:** amyotrophic lateral sclerosis, quality of life, family caregivers, resilience, coping

## Abstract

*Objectives*: Motor neurone disease (MND) progressively damages the nervous system causing wasting to muscles, including those used for breathing. There is robust evidence that non-invasive ventilation (NIV) relieves respiratory symptoms and improves quality of life in MND. Nevertheless, about a third of those who would benefit from NIV decline the treatment. The purpose of the study was to understand this phenomenon. *Design*: A cross-sectional quantitative analysis. *Methods*: Data including age, sex, MND symptomatology, general physical and mental health and psychological measures were collected from 27 patients and their family caregivers at the point of being offered ventilatory support based on physiological markers. *Results*: Quantitative analyses indicated no difference in patient characteristics or symptomatology between those who tolerated (*n* = 17) and those who declined (*n* = 10) NIV treatment. A comparison of family caregivers found no differences in physical or mental health or in caregiving distress, emphasising that this was high in both groups; however, family caregivers supporting NIV treatment were significantly more resilient, less neurotic and less anxious than family caregivers who did not. Regression analyses, forcing MND symptoms to enter the equation first, found caregiver resilience:commitment the strongest predictor of uptake of NIV treatment adding 22% to the 56% explained variance. *Conclusion*: Patients who tolerated NIV treatment had family caregivers who cope through finding meaning and purpose in their situation. Psychological support and proactive involvement for family caregivers in the management of the illness situation is indicated if acceptance of NIV treatment is to be maximised in MND.

## Introduction

Motor neurone disease (MND), also known as amyotrophic lateral sclerosis in some countries, is a neurodegenerative disease of the motor neurones in the brain and spinal cord. There is a clear clinical course of progressive impairment of upper and lower motor neurones leading to weakness and wasting of all muscles, including those used for breathing. Respiratory failure is the primary causes of death in this terminal illness across the globe (Gil et al., 2008; Spataro, Lo Re, Piccoli, Piccoli, & La Bella, 2010; Yang et al., 2011). Symptoms of breathlessness, lack of sleep, feeling tired all the time, morning headaches, lethargy and lack of concentration become increasingly common during disease progression (Kiernan et al., 2011). There is currently no cure for MND, and treatment is based upon management of symptoms towards achieving best possible quality of life.

A major advance in the treatment of MND has been the development of non-invasive ventilation (NIV) to respond to symptoms of chronic hypoventilation and hypoxia. NIV refers to the administration of positive-pressure ventilation from a portable machine via a tight-fitting mask worn over the nose and mouth. Patients needing respiratory support are directed to use NIV to assist breathing during sleep to afford some respite to respiratory muscles and to help restore optimal oxygen and carbon dioxide levels. Use of NIV increases survival rates, which for common forms of MND typically ranges from 2 to 5 years following diagnosis, by 7–18 months in patients with good bulbar function (Bourke, Bullock, Williams, Shaw, & Gibson, 2003; Bourke et al., 2006). Chiò, Calvo, et al. (2012) argue that there is no difference in survival after NIV according to the type of onset symptoms; patients with bulbar symptoms need NIV comparatively earlier than patients with predominant spinal involvement. NIV treatment has additionally been associated with improved patients' sleep, increased participation and activity levels and improved quality of life across various domains (Heiman-Patterson & Miller, 2006; Mustfa et al., 2006; Sundling, Ekman, Weinberg, & Klefbeck, 2009).

MND patients need respiratory tests regularly to detect early signs of impaired performance, and ultimately maximise the efficacy of treatment; UK's National Institute for Health and Care Excellence suggests testing every three months (National Institute for Health and Clinical Excellence [NICE], 2010). Although there is no consensus on the optimal time to introduce NIV treatment (Lo Coco et al., 2012), there is an agreement that NIV is the most effective treatment available for MND where respiratory symptoms are present, and as such NIV should be promoted to patients (Heiman-Patterson & Miller, 2006; Piepers et al., 2006). Typically, the MND patient's care team will explain treatment options, including the potentials of NIV, and how this treatment may improve respiratory symptoms and prolong life.

Although one may intuitively assume that people with terminal illnesses will accept life-extending treatments that also improve quality of life, many studies indicate that such treatment is actively declined by a significant number of those who would benefit from it. This is true in MND despite patients and their family caregivers being given clear evidence of benefits of NIV (Lo Coco et al., 2012; Mustfa et al., 2006). An important question is, therefore, *why do some people with MND reject NIV treatment?*

The rationale for considering that family caregivers may affect uptake and compliance of treatments is best seen in models of family caregiving (Cousins, Davies, Turnbull, & Playfer, 2008). These demonstrate that a diagnosis of disease impacts upon not only the recipient of that diagnosis, but also on their family. Critically, family caregiver characteristics and behaviours, particularly their own health, their personality and their coping styles, interact with patient behaviours and predict patient outcomes in addition to a patient's characteristics and symptomatology. In MND, because of its severe impact on physical functioning, patients come to rely on substantial physical assistance with almost every activity of daily living quite soon after diagnosis (Rabkin, Wagner, & del Bene, 2000). Chiò, Calvo, et al. (2012) found that NIV treatment is more frequently tolerated in married patients, who also had longer survival rates after NIV than non-married patients. They go on to argue that family networks are critical to optimal use of clinical treatments. Family caregivers of people with MND spend an average of 11 hours a day caregiving (Krivickas, Shockley, & Mitsumoto, 1997) and experience considerable sleep disturbance (van Teijlingen, Friend, & Kamal, 2001). This manifests in high levels of burden and depression, both of which increase over time and ultimate results in an overall reduction in quality of life over time (Gauthier et al., 2007; Goldstein, Atkins, Landau, Brown, & Leigh, 2006). Mitsumoto and Rabkin (2007) argue that no family caregivers are more challenged than those of MND patients. In MND assisting with treatment is a part of the caregiving role and NIV is not really an option for someone with MND without the availability and support of a caregiver overnight, particularly where physical help is needed with putting on and taking off the mask (Leigh et al., 2003; Sundling et al., 2009).

Extant studies of the impact of NIV treatment on family caregivers provide a mixed picture. Lo Coco et al. (2012) point out that whilst NIV therapy improves quality of life for patients, caregivers' burden could increase. Mustfa et al. (2006), however, argued that increases in caregiver distress following support of NIV treatment are not significant. Similarly, there have been suggestions that treatments that improve quality of life for patients must be good for family caregivers (Jenkinson et al., 2000); however, this too can be contested. For instance, when Trail, Nelson, Van, Appel, and Lai (2003) asked moderately impaired MND patients and their caregivers about future NIV treatment, there was a substantial difference in the number who responded negatively according to whether they were the patient (3%) or the caregiver (32%), highlighting the fact that patients and caregivers do not always hold the same values and attitudes to NIV treatment.

Some insight into the mixed findings for caregiving outcomes can be found in a qualitative study of the experience of NIV treatment. Sundling et al. (2009) illustrate increased stress and anxiety in family caregivers as they take on supporting NIV, through an increase in their ‘job demands’ and a reduction in their sleep. In this study, over time, family caregivers came to a position of ‘embracing the ventilator’ (p. 117), because of benefits for the patient, whom they loved. Nevertheless, the negative impact on family caregiver well-being remained. This study, however, only included patients and family caregivers who tolerated NIV treatment. There has been little consideration of why NIV may be refused and, to date, there have been no robust investigations of the influence of family caregivers on the uptake of NIV treatment. If NIV treatment requires family caregivers that are willing and able to help, then it follows that family caregivers will have an influence on the uptake of NIV treatment. This paper reports a test of this hypothesis.

## Methods

This study was part of a larger three-year longitudinal, prospective study of NIV treatment in MND from NHS neurology and respiratory clinics in Liverpool, UK, 2008–2011. Ethical approval was granted by the Liverpool Local Research Ethics Committee. All MND patients attending these specialist clinics serving north-west England and Wales were invited to participate in the study. From the whole sample of 35 MND patients participating in the longitudinal research, 28 MND patients were offered NIV treatment, on the basis of baseline lung function testing and overnight pulse oximetry and a review of these assessments by a respiratory physician. One patient died soon after his assessment; the remaining 27 patients and their family caregivers were the participant sample for the study reported here. Seventeen of the 27 MND patients went on to accept NIV treatment and 10 declined. A trial of NIV treatment was available to all participants; most decliners took up this opportunity to some extent, before deciding they would not tolerate the treatment and explicitly rejecting it. Demographic details of all participants can be seen in Table 1.

**Table 1.** Participants in study according to treatment type.

	NIV treatment group	Declined NIV
Patient sex	11 males/6 females	7 males/3 females
Caregiver sex	4 males/13 females	3 males/7 females
Patient age	Range 40–75 years Mean 60.41 years (10.30)	Range 40–79 years Mean 66.20 years (11.17)
Caregiver age	Range 44–82 years Mean 57.56 years (11.70)	Range 43–77 years Mean 65.88 years (10.45)
Patient–caregiver relationship	11 spouses/partners 3 siblings 2 offsprings 1 parent	9 spouses/partners 1 offspring

To investigate variables that predict uptake of NIV treatment, quantitative data collected from patients and family caregivers directly before the NIV decision date were analysed. Measures used for patients were:

*Revised ALS-Functional Rating Scale* (ALS-FRS-R; Cedarbaum et al., 1999). This 12-item five-point scale assesses the severity of disability in MND. Scale scores range from 0 (severe impairment) to 48 (normal functioning).

*Amyotrophic Lateral Sclerosis Assessment Questionnaire* (ALSAQ-40; Jenkinson, Fitzpatrick, Brennan, Bromberg, & Swash, 1999). Patients indicate the frequency of difficulties they may have experienced during the last two weeks in specified activities of daily living using a five-point Likert scale. Scores range from 0 (normal functioning) to 4 (permanent issue) for each item. The answers to the 40 items gives an overall standardised index score and scores for five discrete subscales (eating and drinking (3 items), communication (7 items), activities of daily living and independence (10 items), physical mobility (10 items) and emotional well-being (10 items)).

*MND Dyspnoea Rating Scale* (Dougan, O'Connell, Thornton, & Young, 2000). A 16-item five-point frequency scale assesses the experience of breathing difficulties. Scores range from 0 to 64, where the higher the score, the more severe the problem. There are also three subscales measuring subjective experience of dyspnoea (5 items), emotional aspects (8 items) and mastery of breathing difficulties (3 items).

*Beck Depression Inventory-II* (Beck, Steer, & Brown, 1996). Twenty-one items each scored on a scale value 0–3. Scores range from 0 to 63 and indicate 0–13: minimal depression; 14–19: mild depression; 20–28: moderate depression; and 29–63: severe depression.

*Beck Hopelessness Scale* (Beck, Weissman, Lester, & Trexler, 1974). Items assess feelings about the future, loss of motivation and expectations in a true/false format to provide an overall measure of hopeless. We followed the recommendation of Abbey, Rosenfeld, Pessin, and Breitbart (2006) to use only 13 of the original 20 questions with terminally ill patients.

*Epworth Sleepiness Scale* (ESS; Johns, 1991). Using eight different situations, the ESS asks people to subjectively rate, on a four-point scale (0–3), their chance of dozing off or falling asleep during the day. Their ESS score is the sum of responses, where the higher the score (range 0–24), the higher the level of daytime sleepiness.

Anxiety and depression was assessed in both patients and family caregivers using:

*Hospital Anxiety and Depression Scale* (HADS; Zigmond & Snaith, 1983). Caregiver anxiety and depression was measured with the 14-item HADS. Each item is scored on a 0–3 frequency scale, where high scores indicate greater anxiety or depression. For patients, we used a modified 12-item version in line with the observation that two items (D8 and A11) were unreliable in MND (Gibbons et al., 2011).

Measures used for family caregivers were:

*SF-36v2* (Ware, Kosinski, & Dewey, 2000). A generic health survey comprising 36 items requiring self-assessment of physical health and mental health across the eight domains and four subscales. The survey uses norm-based scoring to allow meaningful comparisons between the domains and subscales.

*Caregiving Distress Scale* (CDS; Cousins, Davies, Turnbull, & Playfer, 2002). A 17-item five-point frequency scale measuring overall distress and five conceptually distinct aspects of caregiving distress (impact on relationships (4 items), impact on social life (3 items), emotional burden (4 items), care-receiver demands (3 items) and personal consequences (3 items)). Higher scores are associated with greater distress.

*Neuroticism* (N; Costa & McCrae, 1992). Dispositional neuroticism was measured with the 12-item five-point N scale from NEO-FFI-R. This variable has previously been found to be an important predictor of caregiving distress (Cousins, 1997) and job satisfaction (Levin & Stokes, 1989). Higher scores indicated greater neuroticism.

*Resilience* (Bartone, Ursano, Wright, & Ingraham, 1989). Resilience represents the characteristic way that people approach and cope with life events (Kobasa, 1979). Resilience is described in terms of three related tendencies: commitment, where behaviour is influenced by the meaning and purpose seen in a situation; control, the ability to make one's own choices in a situation; and challenge, the tendency to perceive life events as opportunities for development, rather than threats. The scale comprises 45 statements each scored 0–3 dependent upon the extent to which the statement is true. Each of the three subscales has 15 items. Higher scores indicate greater resilience in each domain.

## Results

### Is there a difference in patient variables between NIV and no-NIV families?

Of the patient–caregiver families who tolerated NIV were 11 patients with limb-onset and 6 patients with bulbar onset and the no-NIV families comprised 7 MND patients with limb-onset and 3 patients with bulbar onset. An independent samples *t*-test confirmed that this small difference in balance of type of symptoms between the two groups was not significant (*t* = −.56, *p* = .58). Dominant symptom at onset did not differ between those who tolerated NIV and those who did not.

As can be seen in Table 2, there was no difference in disease characteristics at the time of being offered NIV treatment between those patients who went on to accept NIV and those who declined the treatment. Patient symptom variables in those with the potential to benefit from NIV treatment do not predict uptake of the treatment.

**Table 2.** Patient variables means and SDs according to treatment group: NIV (*n* = 17), decliners (*n* = 10).

Variable	Group	Mean	SD	*t*	*p*
Patient age at assessment	NIV	60.41	10.30	1.37	.18
	Decliners	66.20	11.17		
Duration of illness (months)	NIV	28.71	50.94	−0.37	.73
	Decliners	22.00	22.00		
ALS-FRS-R	NIV	28.88	8.70	−0.28	.17
	Decliners	27.90	9.23		
ALSAQ-40 total	NIV	74.59	41.30	0.61	.54
	Decliners	84.40	37.79		
• Eating and drinking	NIV	32.84	28.40	0.39	.70
	Decliners	37.50	32.21		
• Communication	NIV	50.63	38.01	0.19	.85
	Decliners	53.57	41.00		
• ADL and independence	NIV	43.52	26.85	1.05	.31
	Decliners	56.00	34.56		
• Physical mobility	NIV	50.88	35.24	0.98	.34
	Decliners	64.50	34.66		
• Emotional well-being	NIV	30.31	23.41	1.42	.17
	Decliners	44.72	25.93		
MND Dyspnoea Rating Scale	NIV	20.47	10.92	0.87	.39
	Decliners	24.11	8.28		
• Subjective dyspnoea	NIV	5.71	4.36	1.20	.24
	Decliners	8.11	5.75		
• Emotion	NIV	11.94	6.96	0.76	.46
	Decliners	14.00	5.85		
• Mastery	NIV	3.00	2.91	−.95	.35
	Decliners	2.00	1.58		
Beck Depression Inventory-II	NIV	15.18	10.26	0.48	.63
	Decliners	17.00	6.42		
Beck Hopelessness Scale	NIV	4.56	3.69	0.71	.49
	Decliners	5.67	3.85		
Patient anxiety	NIV	4.35	3.37	0.10	.92
	Decliners	4.50	3.92		
Patient depression	NIV	4.06	3.85	0.64	.53
	Decliners	5.00	3.40		
Epworth Sleepiness Scale	NIV	8.29	4.41	−1.24	.23
	Decliners	6.00	5.01		

Note: SD, standard deviation.

### Is there a difference in caregiving distress between NIV and no-NIV families?

As seen in Table 3, independent *t*-tests indicated that NIV caregivers reported significantly less anxiety than no-NIV caregivers (*t* = 2.30, *p* = .03); there was no difference in caregiver depression or on any of the caregiving distress subscales between the two groups. Levels of anxiety and depression were high across all the family caregivers with the overall mean being above accepted clinical cut-off scores (Bjelland, Dahl, Haug, & Neckelmann, 2002). 52% were above the cut-off in terms of anxiety (*M* = 8.37, SD = 3.05) and almost 75% were above the cut-off for depression (*M* = 8.56, SD = 3.65). Levels of caregiver anxiety and depression were above those of the patient sample, even allowing for the revised scoring used in the patient version of the HADS. There was a relationship between caregiver and patient anxiety (*r* = .53, *p* < .01), but no relationship between caregiver and patient depression (*r* = −.15, *p* > .05). Interestingly, the association of patient and caregiver anxiety was stronger in those families that declined NIV treatment (*r* = .79, *p* < .01), than in families that accepted NIV treatment dyads (*r* = .475, *p* = .054). There was no association of patient and caregiver depression according to treatment uptake.

**Table 3.** Family caregiver variables means and SDs according to treatment group: NIV (*n* = 17), decliners (*n* = 10).

Variable	Group	Mean	SD	*t*	*p*
Neuroticism/emotional stability	NIV	16.59	8.52	−2.13	.04*
	Decliners	23.10	5.84		
Hardiness/resilience total	NIV	88.63	13.16	2.71	.01*
	Decliners	73.50	14.99		
Resilience: commitment	NIV	33.31	6.41	2.73	.01*
	Decliners	26.10	6.82		
Resilience: control	NIV	32.00	3.69	2.12	.04*
	Decliners	28.70	4.14		
Resilience: challenge	NIV	23.44	5.44	1.776	.09
	Decliners	18.80	7.91		
Caregiver anxiety (clinical cut-off = 8)	NIV	7.41	3.06	−2.30	.03
	Decliners	10.00	2.36		
Caregiver depression (clinical cut-off = 8)	NIV	7.88	3.97	−1.26	.22
	Decliners	9.70	2.87		
Caregiving Distress Scale (range 0–68)	NIV	26.80	12.39	−0.51	.61
	Decliners	23.94	14.81		
Relationship distress (range 0–16)	NIV	3.40	3.13	0.29	.77
	Decliners	3.82	3.93		
Emotional burden (range 0–16)	NIV	6.10	3.32	−0.55	.59
	Decliners	5.35	3.43		
Care-receiver demands (range 0–12)	NIV	3.30	3.62	0.30	.77
	Decliners	3.71	3.33		
Impact on social life (range 0–12)	NIV	7.90	2.85	−1.09	.29
	Decliners	6.41	3.73		
Personal cost (range 0–12)	NIV	6.10	3.28	−1.20	.24
	Decliners	4.65	2.89		
Physical health summary	NIV	52.51	11.08	1.68	.11
	Decliners	44.92	11.39		
Mental health summary	NIV	40.61	13.94	0.669	.51
	Decliners	36.79	14.55		
Physical functioning	NIV	49.73	7.66	1.86	.08
	Decliners	41.35	13.95		
Role-physical	NIV	49.55	7.66	1.57	.14
	Decliners	42.07	13.84		
Bodily pain	NIV	48.96	12.31	1.33	.20
	Decliners	41.60	15.90		
General health	NIV	49.87	8.76	0.91	.37
	Decliners	45.97	13.64		
Vitality	NIV	46.32	11.04	0.83	.41
	Decliners	42.37	13.30		
Social functioning	NIV	46.28	10.90	1.79	.10
	Decliners	35.97	16.19		
Role emotional	NIV	41.48	11.52	0.61	.55
	Decliners	38.26	15.27		
Mental health	NIV	43.39	13.23	1.09	.29
	Decliners	36.84	17.82		

There was no difference between the two treatment groups in terms of variables on the Caregiving Distress Scale. Both groups exhibited high levels of distress, particularly in relation to Impact on Social Life. All 27 MND family caregivers endorsed at least one aspect of distress although this was clearly not a direct determinant of uptake of NIV treatment.

### Is there a difference in caregiver health status between NIV and no-NIV families?

There was no difference in family caregiver health status between those who were involved in NIV treatment and those who were not (see Table 3). There was a trend towards the physical functioning being worse in decliner caregivers than those supporting NIV. An additional multivariate analysis of variance, co-varying for caregiver age, indicated that this was not simply a reflection of the decliner caregivers being a little older (*F* = 1.99, *p* > .05). Using SF-36v2 tables representative of the mean age of the caregiving sample (60.33 years, range = 43–82), the normalised mean scores reveals that both groups of MND caregivers were below the 50th percentile for this subscale and for all of the other seven subscales. The two summary measures similarly showed that there was no significant difference between the two groups and that caregiver mental health was very poor.

### Is there a difference in caregiver personality and coping style between NIV and no-NIV families?

There was a significant difference in caregiver neuroticism between caregivers supporting NIV treatment and those who were not (*t* = −2.13, *p* < .05). As a group, decliner caregivers were significantly more emotionally unstable than caregivers who tolerated NIV treatment. There was also a significant difference in caregiver resilience; NIV caregivers were significantly more resilient than no-NIV caregivers (*t* = 2.71, *p* = .01), and this pattern was also seen in two of the subscales: commitment (*t* = 2.73, *p* = .01) and control (*t* = 2.12, *p* < .05). There was also trend towards a difference in challenge (*t* = 1.78, *p* = .09). Caregiver personality and coping style was significantly different between the two NIV treatment groups, which strongly suggests that family caregiver variables affect the uptake of NIV treatment.

### What are the key variables that predict uptake of NIV?

To assess the relative contribution of important variables in the use of NIV treatment in MND, a linear regression analysis was performed. First critical MND functioning factors were forced to enter the equation, followed by those caregiving variables which differed between the two groups. When patient functioning variables ALS-FRS-R total, ALSAQ-40, MND dyspnoea rating, ESS, age, depression and anxiety were first forced to enter the equation, together these explained 33.6% of the variance in uptake in NIV treatment (*R* = .579; *R* ^2^ = .336). A separate stepwise regression indicated that none of these MND patient variables was individually significant, and adjusted *R* ^2^ = .004. Then caregiver anxiety, neuroticism, resilience control and resilience commitment were added for a stepwise regression. Caregiver resilience commitment was the only additional variable to enter the equation adding almost 22% to the equation (*R* = .751; *R* ^2^ = .564); adjusted *R* ^2^ = .303. The hypothesis that family caregivers influence uptake of NIV was supported.

## Discussion

This study compared MND patients and their family caregivers according to the uptake of NIV treatment, to address the question of what predicts acceptance of NIV treatment in patients who have been offered it on the basis of impaired respiratory function tests. Consecutive recruitment to this prospective study provided a context where over a third of patients did not accept NIV treatment that had the propensity to both improve their quality of life and extend their life. This level of uptake of treatment is similar to other studies (e.g. Mustfa et al., 2006). Our analyses confirmed there was no differences in MND symptomatology between those patients who tolerated NIV and those that did not; similarly, there was no difference in caregiving distress, indicative of no difference in ‘job demands’. However, there was a strong caregiver influence between the two treatment groups in terms of caregiver dispositional and coping style variables. The key predictor of uptake of NIV treatment was caregiver commitment: resilience that is influenced by the meaning and purpose seen in a situation. Family caregivers of those who tolerated NIV treatment scored significantly higher on this variable than those who declined NIV treatment, and caregiver commitment was able to explain a significant proportion of the variance between the two groups, even after accounting for MND symptomatology, which was forced into the analysis as the primary reason for NIV treatment.

‘How can we maximise the benefit of NIPPV on survival and quality of life in the patient … ?’ (Heiman-Patterson & Miller, 2006, p. 737) is a pertinent question, if one accepts that all patients who are offered NIV treatment can benefit from it (Miller et al., 2009). This question is partially answered by this research, in so far as lower caregiver resilience in terms of commitment is predictive of NIV treatment being declined and hence the patient not benefiting from improvements to quality of life. Low commitment is conceptualised as a lack of resilience that is underpinned by not seeing meaning and purpose in a situation (Bartone et al., 1989).

Peters, Fitzpatrick, Doll, Playford, and Jenkinson (2012) assert that a key problem for MND family caregivers is not feeling sufficiently involved in the planning of care. Over three quarters of the 434 family caregivers who participated in their survey responded that they perceived a lack of value of their experiences from health and social care services. And more specifically related to NIV treatment, Kaub-Wittemer, von Steinbüchel, Wasner, Laier-Groeneveld, and Borasio (2003) found that knowledge of the disease was insufficient in one-third of their sample of 32 family caregivers regarding the need for and efficacy of NIV treatment. NICE (2010) guidelines indicate that families and carers should ask about the availability of training to support breathing problems and NIV treatment. A pre-emptive training package for family caregivers towards enabling them to support NIV treatment may be a better way of maximising the benefits of NIV (Leigh et al., 2003). Accepting that the emphasis of care should be on autonomy and choice, there remains a need for suitable and sufficient knowledge of MND and NIV in order to be able to make an informed choice about going forward with NIV treatment.

Although there was no difference between the two NIV uptake groups in terms of caregiver depression and caregiving distress, levels of depression and distress were nevertheless high. In this sample, about three quarters of the sample scored above clinical cut-offs for depression. These results are not peculiar to this research study (Peters et al., 2012), and it has been clearly pointed out that psychological interventions to reduce caregiver distress and anxiety are beneficial for patient outcomes (Cousins et al., 2002; Murphy, Felgoise, Walsh, & Simmons, 2009; Rabkin et al., 2000).

The finding that family caregiving variables influence the uptake of NIV treatment is a new finding, pertinent to the MND illness situation. This result adds to the growing literature on the importance of family caregiver involvement for maximising the efficacy of health interventions. There is evidence that participation of family caregivers is essential for ensuring compliance to treatment in cancer, heart disease, Parkinson's disease and dementia (Brodaty & Green, 2002; Clark et al., 2012; Davies, Cousins, Turnbull, & Playfer, 1999; Glajchen, 2004). Clark et al. (2012) similarly questioned why up to half of people referred to coronary heart disease (CHD) programmes do not participate. Their systematic review of 90 studies in the area of rehabilitation in CHD found that level of family support was highly predictive of attendance and level of participation in treatment programmes, and thus they recommend intervention to increase participation should harness family members as a particularly promising and effective means by which to support attendance.

A recent update of a survey of NIV use in the UK (O'Neill et al., 2012) indicates that whilst the number of new cases of MND has remained stable since their study published 10 years earlier (Bourke et al., 2003), referral to NIV treatment has more than doubled and successful initiation on NIV treatment trebled. The survey findings indicate that clinics with the highest rates of NIV uptake were those that routinely monitor respiratory function. This finding strongly supports the efforts made to promote NIV treatment, but it does not provide sufficient confidence to consider that further intervention is not required to maximise the uptake of NIV treatment, such as involving family caregivers more proactively, as the evidence in this study allows us to propose. There are, of course, other predictors and barriers that also influence the recommendation of and uptake of NIV treatment. Various studies suggest that bulbar impairment could negatively affect NIV tolerance and compliance (Lo Coco et al., 2012; Miller et al., 2009). In this study, one-third of the sample were bulbar onset patients; however, type of onset did not predict tolerance to NIV. Moreover, two-thirds of the bulbar patients accepted NIV treatment. Other researchers have similarly asserted that bulbar symptoms do not prevent NIV treatment (Chiò, Calvo, et al., 2012; Leigh et al., 2003), which is indicative that NIV is an effective treatment for all MND patients whose respiratory system is impaired to the extent outlined by Miller et al. (1999). Chiò, Ilardi, et al. (2012) has also illustrated how neurobehavioural dysfunction, cognitive impairment and concomitant fronto-temporal dementia in patients serve as a barrier to the successful use of NIV treatment.

In conclusion, the results of this study clearly demonstrate that family caregiver variables, and particularly caregiver resilience, impact upon the uptake of NIV treatment in MND. We recommend that family caregivers should be seen as critically important to maximising the benefit of NIV on survival and quality of life in the patient. A pre-emptive support programme for family caregivers should be a part of the multidisciplinary care package in MND.
